# Lawrence Whalley MD, FRCP (E), FRCPsych

**DOI:** 10.1192/bjb.2024.78

**Published:** 2024-12

**Authors:** David St Clair, Rob Wrate

Emeritus Professor of Mental Health, University of Aberdeen, UK, formerly Honorary Consultant, NHS Grampian, Aberdeen, UK

**Figure:**
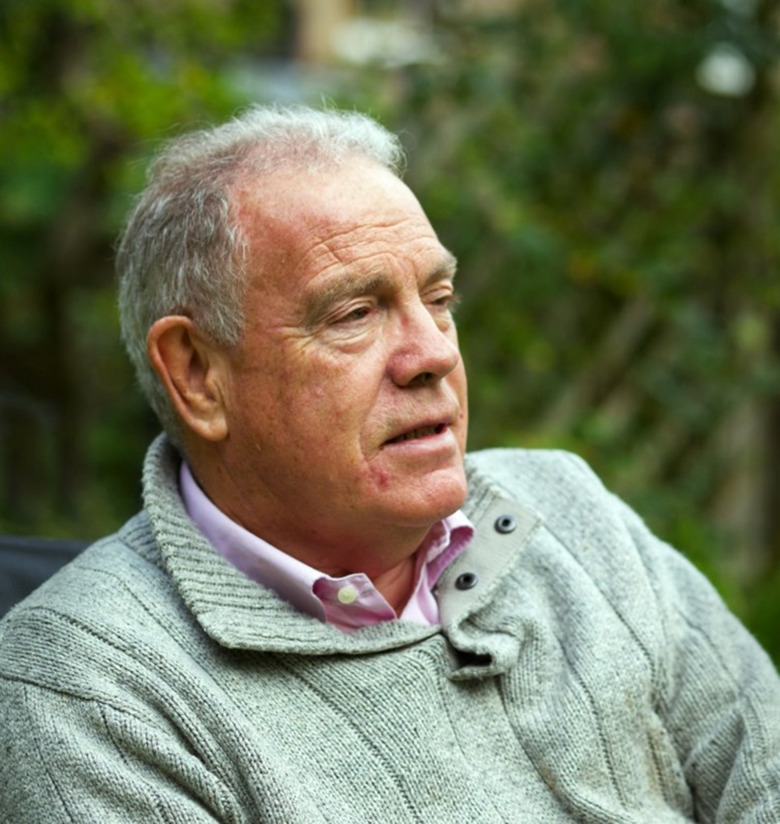

Lawrence Whalley, who died suddenly aged 78 on 11 April 2024, made highly significant contributions over many years to knowledge of the life course of cognitive ageing and dementia. Not long after moving to the Crombie Ross Professorship in Mental Health in 1983, he and his wife, Patricia, co-discovered that in 1932 and 1947 all children in Scotland aged eleven had had their IQs tested under the auspices of the Scottish Council for Research in Education. Furthermore, these long-forgotten records of 150 000 Scottish children had been archived. Thus began a series of landmark and world-famous population-based longitudinal studies. With the key involvement of Edinburgh colleagues, Professor Ian Deary and the late John Starr, Lawrence began systematically to trace these individuals, initially in Aberdeen and then in Edinburgh, and, if still alive, to ask their consent to be re-examined using modern methods. This allowed a life-course approach to understanding their brain health in later life and to measure precisely the amount of cognitive decline experienced by the research participants from early adolescence into late life. They described their findings in a large number of papers. Most importantly, they discovered how a lower initial IQ aged 11 predicted both increased life-time morbidity and mortality (e.g. by lung cancer) independent of social class, as well as more decline with age in higher cognitive functioning. Other factors such as deprivation, physical fitness and personality (e.g. lack of ‘openness to experience’) were additional major individual risk factors that seemed to influence cognitive decline. Later Lothian Birth Cohort studies in Edinburgh were able to identify ApoE epsilon 4 allele and white matter hyperintensity signals as significant genetic and imaging risk factors.

Prior to his appointment in Aberdeen, when based at the MRC Brain Metabolism Unit at the Royal Hospital of Edinburgh, Lawrence with fellow psychiatric clinician scientist Dr Janice Christie directed a neuroendocrine psychiatric research programme. It generated multiple excellent scientific publications. Unfortunately, like so many psychiatric biomarker discovery efforts right up to today, little emerged of clinical utility. This programme did, however, provide Lawrence with an introduction to the critical need for sound research methodology. He realised the importance of having sufficient statistical power together with careful characterisation of the clinical phenotype. Long afterwards he spoke appreciatively of his colleagues, especially key mentors in neurobiology, epidemiology and statistics – respectively, Professors George Fink, Norman Kreitman and Ralph Maguire.

During this period, Lawrence Whalley applied his carefully honed skills in a different field. He became interested in the neurobiology of human intellectual decline. He observed that cases of early-onset dementia were not randomly distributed but seemed to cluster. This raised the possibility that adverse public health exposures might be responsible; the question then arose as to which might be the critical exposures and how long they might precede the development of the early onset dementia phenotypes themselves.

Born in Lancashire, on 3 March 1946, one of five children to James Whalley, a mechanical engineer, and his wife, Florence, Lawrence Whalley was educated at St Joseph's College in Blackpool, where his young enquiring mind was inspired by the teaching brothers. He received his undergraduate medical education at the University of Newcastle and his postgraduate psychiatric training at the Royal Edinburgh Hospital. In 1976, he was awarded an MD. After the junior psychiatric training rotation, he also received his higher training in psychiatry in Edinburgh.

Lawrence was elected Fellow of both the Royal College of Psychiatrists and the Royal College of Physicians in Edinburgh. In 2001, he was awarded a prestigious 5-year Wellcome Trust Professorial Fellowship; this allowed him to pursue his academic work full time. Lawrence was immensely productive with over 300 peer-reviewed publications and multiple book chapters. More than a hundred of the publications followed his retirement in 2008. He also wrote for a lay readership, for example, *The Ageing Brain* (2001, 2004), which he dedicated to his family.

Lawrence married his first wife, Patricia, as a medical student in 1969, and was father to their three daughters. After their divorce, he married Helen Fox in Aberdeen, but remained closely involved with both families until his death. Although a resident of Scotland for more than 50 years, Lawrence remained proud of his Lancashire roots, and he never lost his accent. Underlying his direct way of speaking and sometimes brusque manner was a complex emotional man for whom personal relationships were extremely important, most of all with his large circle of family members. He will be remembered not only for his academic dedication and achievements, but for his character: full of energy, generosity for and loyalty to friends, family members, and colleagues. His intellectual curiosity continued undiminished to the end; he was writing and reviewing papers right up to his sudden unexpected death at home in Edinburgh.

He is survived by Patricia and Helen, his three daughters, Charlotte, Amanda and Elizabeth, three step-children, Nicola, Christina and Ronan, and six grandchildren.

